# Comprehensive literature review on the application of the otological surgical planning software OTOPLAN® for cochlear implantation

**DOI:** 10.1007/s00106-023-01417-4

**Published:** 2024-06-11

**Authors:** Franz-Tassilo Müller-Graff, Björn Spahn, David P. Herrmann, Anja Kurz, Johannes Völker, Rudolf Hagen, Kristen Rak

**Affiliations:** Department of Oto-Rhino-Laryngology, Plastic, Aesthetic and Reconstructive Head and Neck Surgery and the Comprehensive Hearing Center, University of Wuerzburg, Josef-Schneider-Straße 11, 97080 Wuerzburg, Germany

**Keywords:** Anatomy based fitting, Computed tomography-based software ear/cochlear, Imaging modalities (MRI, computer tomography [flat-panel volume CT]), Cochlear duct length, Computer simulation, Anatomiebasiertes Fitting, Computertomographie-basierte Software Ohr/Cochlea, Bildgebungsmodalitäten (MRT, Computertomographie [flat-panel volume CT]), Cochleäre Länge, Computersimulation

## Abstract

**Background:**

The size of the human cochlear, measured by the diameter of the basal turn, varies between 7 and 11 mm. For hearing rehabilitation with cochlear implants (CI), the size of the cochlear influences the individual frequency map and the choice of electrode length. OTOPLAN® (CAScination AG [Bern, Switzerland] in cooperation with MED-EL [Innsbruck, Austria]) is a software tool with CE marking for clinical applications in CI treatment which allows for precise pre-planning based on cochlear size. This literature review aims to analyze all published data on the application of OTOPLAN®.

**Materials and methods:**

The Preferred Reporting Items for Systematic Reviews and Meta-Analyses (PRISMA) guidelines were applied to identify relevant studies published in the PubMed search engine between January 2015 and February 2023 using the search terms “otoplan” [title/abstract] OR “anatomy-based fitting” [title/abstract] OR “otological software tool” [title/abstract] OR “computed tomography-based software AND cochlear” [title/abstract].

**Results:**

The systematic review of the literature identified 32 studies on clinical use of OTOPLAN® in CI treatment. Most studies were reported from Germany (7 out of 32), followed by Italy (5), Saudi Arabia (4), the USA (4), and Belgium (3); 2 studies each were from Austria and China, and 1 study from France, India, Norway, South Korea, and Switzerland. In the majority of studies (22), OTOPLAN® was used to assess cochlear size, followed by visualizing the electrode position using postoperative images (5), three-dimensional segmentation of temporal bone structures (4), planning the electrode insertion trajectory (3), creating a patient-specific frequency map (3), planning of a safe drilling path through the facial recess (3), and measuring of temporal bone structures (1).

**Conclusion:**

To date, OTOPLAN® is the only DICOM viewer with CE marking in the CI field that can process pre-, intra-, and postoperative images in the abovementioned applications.

## Abstract (de)

**Hintergrund:**

Die Größe der menschlichen Cochlea, gemessen am Durchmesser der Basalwindung, schwankt zwischen 7 und 11 mm. Im Rahmen einer Hörrehabilitation durch ein Cochleaimplantat ist diese für die individuelle Zuordnung der Frequenzbänder und die Wahl der Elektrodenlänge von Bedeutung. OTOPLAN® (CAScination AG [Bern, Schweiz] in Kooperation mit MED-EL [Innsbruck, Österreich]) ist ein Softwaretool mit CE-Kennzeichnung für klinische Anwendungen in der Cochleaimplantat(CI)-Behandlung, welches die Vorplanung auf Grundlage der cochleären Größenparameter durchführt. Ziel dieser Literaturübersicht ist es, alle veröffentlichten Studien über die Anwendung von OTOPLAN® zu erfassen.

**Materialien und Methoden:**

Die PRISMA-Richtlinien (Preferred Reporting Items for Systematic Reviews and Meta-Analyses) wurden angewandt, um relevante Studien zu identifizieren, die zwischen Januar 2015 und Februar 2023 in der Suchmaschine PubMed veröffentlicht wurden (unter Verwendung der Suchbegriffe „otoplan“ [Titel/Abstract] OR „anatomy-based fitting“ [Titel/Abstract] OR „otological software tool“ [Titel/Abstract] OR „computed tomography-based software AND cochlear“ [Titel/Abstract]).

**Ergebnisse:**

Bei der systematischen Durchsicht der Literatur wurden 32 Studien über den klinischen Einsatz von OTOPLAN® bei der CI-Behandlung gefunden. Die meisten Studien wurden von deutschen Arbeitsgruppen publiziert (7 von 32), gefolgt von Italien (5), Saudi-Arabien (4), USA (4) und Belgien (3). So stammten je 2 Studien aus Österreich und China, gefolgt von jeweils 1 Studie aus Frankreich, Indien, Norwegen, Südkorea und der Schweiz. In den meisten Studien (22) wurde OTOPLAN® zur Beurteilung der Cochleagröße verwendet, gefolgt von der Visualisierung der Elektrodenposition anhand postoperativer Bilder (5), der dreidimensionalen (3-D-)Segmentierung der Felsenbeinstrukturen (4), der Planung der Elektrodeneinführungstrajektorie (3), der Erstellung einer patientenspezifischen Frequenzbandzuordnung (3), der Planung eines sicheren Bohrpfads durch den Recessus facialis (3), und der Messung von Felsenbeinstrukturen (1).

**Schlussfolgerung:**

OTOPLAN® ist bisher der einzige DICOM-Viewer mit CE-Kennzeichnung im CI-Bereich, der prä-, intra- und postoperative Bilder mit den genannten Anwendungen verarbeiten kann.

## Introduction

### Design of a cochlear implant

Cochlear implantation (CI) is a proven technology that has been used in clinical routine to restore hearing in sensorineural hearing loss for more than 40 years [1]. To date (at the time of drafting this article), a total of 900,000 cochlear implants have been successfully implanted [54]. A cochlear implant consists of an externally worn sound processor and an implantable electronic circuit encased in a titanium case, along with an intracochlear electrode. The sound signal—recorded in the sound processor—is converted into frequency-specific digital signals that are transmitted to the implantable electronics via an inductive connection. The implantable electronics transduces these frequency-specific digital signals into frequency-matched electrical pulses, which are then delivered to the cochlea via an intracochlear electrode array placed longitudinally in the scala tympani (ST). The neural elements in the cochlea are arranged tonotopically, with higher frequencies at the basal end, lower frequencies at the apical end, and intermediate frequencies in between. These neural elements pick up the electrical signal and transmit it to the auditory nerve, which carries it to the auditory cortex where it is perceived as sound [14].

### Prerequisites for the success of the surgery

Surgical placement of the CI electrode in the cochlea to create an effective electrode–neural interface is one of the key factors for successful CI treatment [15]. The general variation of the cochlear size allows for different insertion depths of one and the same electrode [21]. It has been reported that a sufficient congruity in length between the electrode and the ST results in a good match in pitch perception between the naturally hearing side and the CI-implanted side in unilaterally deaf individuals [50]. It should be noted that these data were collected only with electrodes from one CI manufacturer (MED-EL, Innsbruck, Austria) and in a small sample size. A longer electrode covering most of the cochlea also produces better hearing results than a short electrode covering only the basal turn of the cochlea in profoundly deaf persons [8, 9, 20, 25, 45]. This can be safely and consistently achieved in any CI candidate if the cochlear size is known preoperatively, which helps CI surgeons select an electrode with the appropriate length.

Anatomical variations in size and shape of the human cochlea have been extensively reported in the literature. In 2005, the French radiologist Dr. Bernard Escude reported that the basic cochlear parameter, the basal turn diameter (A value) in the so-called cochlear view (i.e., the coronal oblique view), can predict the cochlear duct length (CDL) along the outer lateral wall (LW) from the entrance of the round window (RW) to any insertion depth (CDL_LW_; [19]). However, it must be pointed out that there is a considerable interrater variance in this formula [7]. Since then, there have been several reports of fine-tuned mathematical equations to predict CDL along the basilar membrane (BM; CDL_BM_) or the organ of Corti (OC; CDL_OC_), which is more relevant because the straight lateral wall electrode type would sit directly under the BM or OC [32, 52]. The Greenwood frequency function also incorporates the CDL along the OC to obtain the patient-specific frequency map [56].

Accurate measurement of cochlear size helps to (a) estimate the CDL, (b) create a patient-specific frequency map, (c) determine the insertion depth at which residual hearing begins at the apical end of the cochlea, (d) match an electrode length to the CDL, and (d) determine residual hearing. The accuracy and reproducibility of cochlear size measurement by different observers plays a critical role in the overall success of using cochlear size measurement in clinical research.

With the launch of OTOPLAN® (Cascination AG, Bern, Switzerland) in 2018 and CE (*Conformité Européenne*) marking, a dedicated software tool for CI preplanning was introduced that (a) simplifies the measurement of cochlear size, (b) enables visualization of patient-specific frequency maps, (c) simulates the best-fitting electrode length, and (d) controls the postoperative position of an inserted electrode when evaluating postoperative imaging. Our own experience with the clinical application of OTOPLAN® software motivated us to search the literature to determine how effectively OTOPLAN® was used in the clinical CI setting to date.

## Methods

The aim of this review was to identify the clinical applications of OTOPLAN® in the CI field.

### Search strategy

The review was conducted according to the Preferred Reporting Items for Systematic Reviews and Meta-analyses (PRISMA) guidelines [40], using PubMed as the search engine. Articles published from the beginning of January 2015 to the end of February 2023 were included in the search. This period marks the time after the introduction of OTOPLAN® as a research tool in 2015.

### Study selection

The most relevant search articles were extracted by one of the authors using predefined search terms. Broad search criteria were used to include as many published articles as possible. The search terms were: (“otoplan” [Title/Abstract] OR “anatomy-based fitting” [Title/Abstract] OR “otological software tool” [Title/Abstract] OR “computed tomography-based software AND cochlear” [Titel/Abstract]). Review articles containing the term *OTOPLAN*® in the abstract were excluded from this systematic literature review.

Titles and/or abstracts were thoroughly screened manually to identify studies that met the inclusion and exclusion criteria. Two authors (FTMG and KR) independently reviewed the articles. The information extracted from the relevant articles was used to fill a predefined Excel (Microsoft Corporation, Redmond, WA, USA) spreadsheet. The table included the PubMed ID, authors of the article, year of publication, country of origin, type of study, objective of the study using OTOPLAN®, number of study participants, anatomy of the temporal bone analyzed, and age of the CI participants. Disagreements between reviewers about the data collected were resolved by mutual discussion and consensus. These concerned in particular the assignment of individual studies to the various applications of the software, since some studies dealt with several functions at the same time.

## Results

The search was initiated on 20 February 2023 to include all studies that used OTOPLAN® at the time of drafting this article. All identified studies reported the successful use of OTOPLAN®.

### Description of the studies

**Fig. 1:**
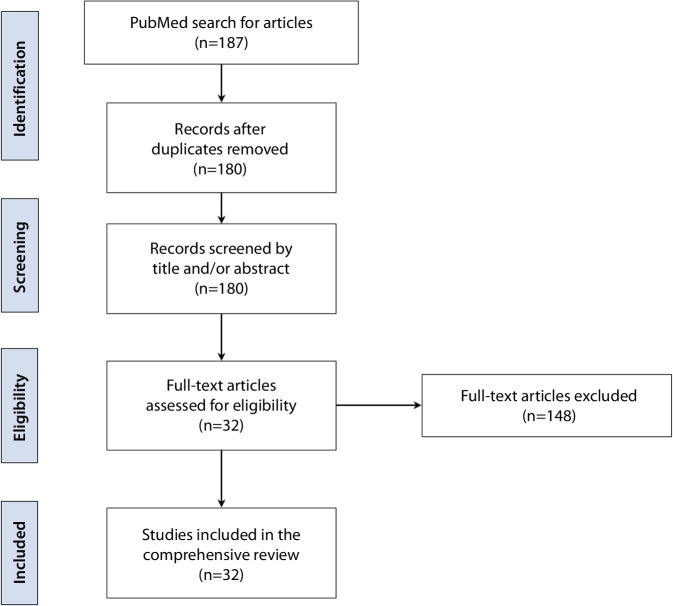
Flowchart depicting the literature review process using the guidelines of Preferred Reporting Items for Systematic Reviews and Meta-analyses (PRISMA)

In total, 187 relevant studies initially met the inclusion and exclusion criteria. Figure 1 shows a flowchart listing the number of studies identified at each step according to the PRISMA guidelines. After removing duplicates, a total of 148 studies were excluded from the remaining 180 studies after screening the title and/or abstract. Thus, a total of 32 studies remained in the final systematic review.

### Demographics

Table 1 shows the demographic data of the studies collected from the 32 relevant publications. Overall, 23 studies were retrospective, two were cadaveric studies, two were case reports, two were prospective, one was a clinical trial, and for the remainder, the type of study was not specified. The studies were published from multiple geographic locations from different continents. Seven studies were from Germany, five from Italy, four each from Saudi Arabia and the United States, three from Belgium, two each from Austria and China, and one each from France, India, Norway, South Korea, and Switzerland.

**Table 1:** Demographic data collection from the 31 identified studies

PMID	Author	Country of origin	Study design	Aim with OTOPLAN®	Number of patients	Anatomies analyzed	Age of patients
26736914	Lu et al. 2015 [47]	Switzerland	Cadaveric study	Segmenting the facial nerve from clinical CT images	5	Normal anatomy	Cadaveric heads
30531645	Lovato et al. 2019 [34]	Italy	Case report	Preoperative surgical planning in a post-meningitis ossification case	1	Ossified cochlea	46 years
32569151	Lovato et al. 2020 [35]	Italy	Prospective	Surgical planning of CI in patients with advanced otosclerosis	5	Far advanced otosclerosis	59.6 years
32209514	Topsakal et al. 2020 [59]	Belgium	Retrospective	Comparison of electrode insertion trajectory for different surgical techniques	Not reported	Normal anatomy	Not reported
32493102	Khurayzi et al. 2020 [28]	Saudi Arabia	Retrospective	Comparison of A‑value measurement between OTOPLAN® and standard DICOM viewer	88	Normal anatomy	1–7 years
32080026	Almuhawas et al. 2020 [3]	Saudi Arabia	Retrospective	Measurement of mastoid thickness and skull width	92	Normal and malformed anatomy	0.5–79 years
34820415	Jablonski et al. 2021 [27]	Norway	Cadaveric study	Access to the RW exclusively with the image-guided robotic system instead of manual drilling into the RW	16	Normal anatomy	Cadaveric heads
33273309	Mlynski et al. [39]	Germany	Retrospective	Measurement of CDL and correlation with postoperative speech performance and with ECAP	53	Normal anatomy	63.6 years
34590531	Cooperman et al. 2021 [13]	USA	Retrospective	Estimation of CDL by measuring the A value of the cochlea	61	Normal anatomy	Adult patients
34050805	Spiegel et al. 2021 [55]	Germany	Retrospective	Estimation of CDL	180	Normal anatomy	6.5–90.3 years
33710146	Chen et al. 2021 [11]	China	Retrospective	Estimation of CDL and comparison with MPR	68	Normal anatomy	0.6–63.3 years
33492059	Cooperman et al. 2021 [12]	USA	Retrospective	Measuring CDL	166	Normal anatomy	65.63 years
33455125	Niu et al. 2021 [43]	China	Prospective	Estimation of CDL and choice of electrode length	26	Normal anatomy	19–71 years
33143454	Andersen et al. 2021 [4]	USA	Retrospective	Segmentation of middle ear and inner ear structures	9	Normal anatomy	3–12 years
32826506	Lee et al. 2021 [31]	South Korea	Retrospective	Measurement of cochlear parameters	51	Normal anatomy	26–112 months 12–468 months 7–91 months
34660683	Auinger et al. 2021 [5]	Austria	Retrospective	Planning the drilling trajectory from the skull surface to the cochlear entrance while safely traversing the facial recess	50	Normal anatomy	51 ± 23
36351223	Kurz et al. 2022 [29]	Germany	Retrospective	Application of anatomy-based fitting in experienced CI users	3	Normal anatomy	57, 57, 38 years
36544941	Dhanasingh et al. 2022 [16]	Austria	Not reported	Systematic visualization of the inner ear in both cochlear view (oblique coronal plane) and mid-modiolar section (axial plane) and following three sequential steps simplifies identification of types of inner ear malformations	112	Normal and malformed anatomy	Not reported
34101009	Müller-Graff et al. 2022 [41]	Germany	Retrospective	Visualization of pre- and postoperative secondary reconstructions of flat-panel volume CTs, including estimation of CDL and position of electrode contacts	30	Normal anatomy	64 years
32925847	George-Jones et al. 2022 [22]	USA	Retrospective	Comparison of cochlear size using CT and MRI	21	Normal anatomy	Not reported
36294805	Li et al. 2022 [33]	China	Retrospective	Measurement of cochlear parameters (A, B, and H values)	247	Normal anatomy and EVAS	< 18 years
35970933	Weber et al. 2022 [60]	Germany	Retrospective	Comparison of CT and MRI to cross-check the A‑value measurement	20	Normal anatomy	21–71 years
35386404	Topsakal et al. 2022 [58]	Belgium	Clinical trial	Evaluation of the intraoperative accuracy of robotic middle ear and inner ear access with respect to distance from critical anatomic structures (such as ChT and FN) and intended target	22	21 Normal anatomy and 1 incomplete partition type III	28–83 years
35193850	Ricci et al. 2022 [49]	Italy	Case study	Analysis of CT scans with advanced otosclerosis and measurement of cochlear parameters (A, B, and H values)	1	Advanced otosclerosis	73 years
35032205	Di Maro et al. 2022 [17]	Italy	Retrospective	Changing from the default frequency map to patient-specific frequency map	10	Normal anatomy	14.3–78.7 years
34538852	Dutrieux et al. 2022 [18]	France	Retrospective	Evaluation of CDL, insertion angle, and insertion depth	106	Normal anatomy	61 years
34131770	Mertens et al. 2022 [37]	Belgium	Retrospective	Measurement of cochlear size and application of a patient-specific frequency map	39	Normal anatomy	17–81 years
36436080	Thimsen et al. 2022 [57]	Germany	Retrospective	Evaluation of CDL and insertion depth	19	Normal anatomy	18–75 years
36514425	Bhavana et al. 2022 [6]	India	Retrospective	Evaluation of CDL and insertion depth	26	Normal anatomy	2–15 years
36836405	Alahmadi et al. 2023 [2]	Saudi Arabia	Retrospective	Measurement of cochlear parameters	21	EVAS	13.81 years
36617441	Müller-Graff et al. 2023 [42]	Germany	Retrospective	Evaluation of the accuracy of radiological prediction of postoperative electrode position based on preoperative imaging	10	Normal anatomy	58 years
36609169	Hagr et al. 2023 [24]	Saudi Arabia	Not reported	Determining the best electrode trajectory in CI surgery using the reconstructed 3D model and investigation of the surgical removal of the retrofacial approach as a direct approach to the RW	25	Normal anatomy	6.8 ± 12 years

*3D* three-dimensional, *CDL* cochlear duct length, *ChT* chorda tympani, *CI* cochlear implant, *CT* computed tomography, *EVAS* enlarged vestibular aqueduct syndrome, *FN* facial nerve, *MPR* multiplanar reconstruction, *MRI* magnetic resonance imaging, *RW* round window

### Applications of OTOPLAN®

In the majority of studies OTOPLAN® was applied in normal anatomy. While Ricci et al. [49] and Lovato et al. used it in advanced otosclerosis, Lovato et al. [35] deployed it in post-meningitis ossified conditions. Topsakal et al. applied it in incomplete partition type III malformation, Li et al. [33] and Alahmadi et al. [2] used it for enlarged vestibular aqueduct, and Dhanasingh et al. [16] successfully deployed it in a variety of inner ear malformations.

The main outcomes were (a) visualization of the inner ear and measurement of cochlear parameters on both computed tomography (CT) and ,magnetic resonance imaging (MRI; together: 22 studies); (b) segmentation of the middle ear, inner ear structures, and facial nerve (four studies); (c) surgical planning for the best trajectory of electrode insertion, to preserve the critical anatomic structures and consecutive robotic drilling through the facial recess (six studies); (d) evaluation of postoperative imaging related to electrode position and insertion depth (five studies); (e) reallocating of center frequencies based on a patient-specific frequency map (three studies); and (f) measurement function of temporal bone structures (one study).

#### Cochlear size measurement

**Fig. 2:**
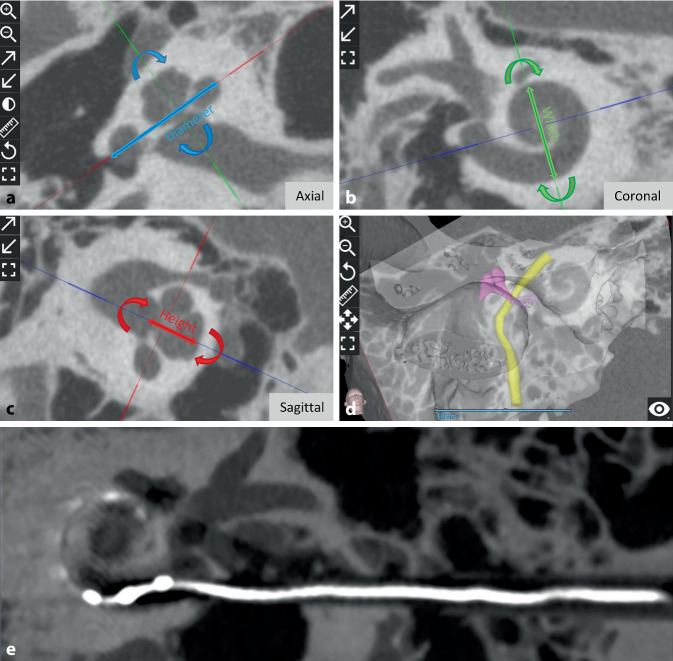
Exemplary representation of the preoperative planning of a cochlear implantation and visualization of an implanted electrode with direct cochlear access pathway. Measurement of the cochlea using the corresponding parameters in the “cochlear view,” generated by rotation around the body axes. Axial view (**a**), coronal view (**b**), sagittal view (**c**), and three-dimensional visualization of the temporal bone, ossicles, and facial nerve (**d**). *Blue double arrow* diameter, *green double arrow* width, and *red double arrow* height. Postoperative image of a direct cochlear access (DCA) pathway with an electrode inside (**e**). (**e** From the open access publication of Jablonski et al. 2021 [27] © Jablonski et al.; CCBY4.0; https://creativecommons.org/licenses/by/4.0/)

Of 32 studies, 22 reported specifically on the measurement of cochlear size at different geographic locations. By rotating the three body planes in imaging, the coronal oblique view, called the “cochlear view,” provides a standard uniform view of the cochlea that can be used to reliably measure cochlear parameters. These are the diameter (A value), width (B value), and height (H value), as shown in Fig. 2a–c, from which the CDL can then be calculated. Table 2 shows the image types used and summarizes the measurement of cochlear size using the A value and CDL in millimeters. Those studies that reported only the CDL without the A value should have measured (but not reported) the A value, as the CDL is estimated from the A value.

**Table 2:** Data on image types and cochlear measurements from the 31 identified studies

Study no.	Author	Pre-/postoperative image	Image type	Imaging modality	A value (mm)	CDL (mm)
1	Lu et al. 2015 [47]	Pre-op	CT	CBCT	–	–
2	Lovato et al. 2019 [34]	Pre-op	CT	TBCT	–	–
3	Lovato et al. 2020 [35]	Pre-op	CT	HRCT	–	32.4
4	Topsakal et al. 2020 [59]	Pre-op	CT	HRCT	8.44 ± 0.4 (7.6–9.3)	–
5	Khurayzi et al. 2020 [28]	Pre-op	CT	HRCT	7.6–10.2	–
6	Almuhawas et al. 2020 [3]	Pre-op	CT	N/A	9.1 ± 0.27	32.5 ± 1.2
7	Jablonski et al. 2021 [27]	Pre- and post-op	CT	CBCT	–	33.44 (29.30–38.25)
8	Mlynski et al. 2021 [39]	Pre-op	CT	HRCT	–	35.00 (SD ± 2.2)
9	Cooperman et al. 2021 [13]	Pre-op	CT	N/A	–	36.2 ± 1.8
10	Spiegel et al. 2021 [55]	Pre-op	CT	N/A	9.33 ± 0.37	34.37 ± 1.5
11	Chen et al. 2021 [11]	Pre-op	CT	N/A	–	32.84 ± 2.0 (29.0–38.1)
12	Cooperman et al. 2021 [12]	Pre-op	CT	CBCT	–	–
13	Niu et al. 2021 [43]	Pre-op	CT	HRCT	–	–
14	Andersen et al. 2021 [4]	Pre-op	CT	N/A	–	–
15	Lee et al. 2021 [31]	Pre-op	CT	HRCT	–	32.40 ± 1.26 34.94 ± 1.20 35.77 ± 1.15
16	Auinger et al. 2021 [5]	Pre-op	CT	HRCT and CBCT	9.3	35.82 ± 1.56
17	Kurz et al. 2022 [29]	Pre- and post-op	CT	fpVCT_SECO_	–	–
18	Dhanasingh et al. 2022 [16]	Pre-op	CT	MSCT	9.0 (8.1–10.1)	–
19	Müller-Graff et al. 2022 [41]	Pre- and post-op	CT	MSCT, fpVCT, fpVCT_SECO_	–	34.5 ± 1.6 (31.2–36.9) 34.6 ± 1.47 (31.5–37.6) 35.84 ± 1.39 (32.9–38.4)
20	George-Jones et al. 2022 [22]	Pre-op	CT and MRI	TBCT and MRI	–	32.7 ± 2.0 (29.4–37.6)
21	Li et al. 2022 [33]	Pre-op	CT	HRCT	8.8 (7.4–9.7)	–
22	Weber et al. 2022 [60]	Pre-op	CT and MRI	fpVCT and MRI	9.31 ± 0.44	36.5 ± 1.59
23	Topsakal et al. 2022 [58]	Pre- and post-op	CT	CBCT	–	–
24	Ricci et al. 2022 [49]	Pre-op	CT	TBCT	–	–
25	Di Maro et al. 2022 [17]	Post-op	CT	HRCT	–	41.37 ± 3.1
26	Dutrieux et al. 2022 [18]	Post-op	CT	MSCT and CBCT	–	34.5 ± 3.5
27	Mertens et al. 2022 [37]	Pre- and post-op	CT	N/A	–	32.96 ± 0.73 (31.0–34.40)
28	Thimsen et al. 2022 [57]	Pre- and post-op	CT	MSCT and fpVCT	–	–
29	Bhavana et al. 2022 [6]	Pre- and post-op	CT	N/A	–	38.12 (34.2–43)
30	Alahmadi et al. 2023 [2]	Pre-op	CT	MSCT	8.36 ± 0.32 (female) 8.82 ± 0.42 (male)	–
31	Müller-Graff et al. 2023 [42]	Pre- and post-op	CT	MSCT, fpVCT, fpVCT_SECO_	–	33.2 ± 2.2 33.9 ± 2.0 34.9 ± 1.8
32	Hagr et al. 2023 [24]	Pre-op	CT	HRCT	–	–

*CBCT* cone-beam computed tomography, *CDL* cochlear duct length, *CT* computed tomography, *fpVCT* flat panel volume computed tomography, *fpVC*_*TSECO*_ secondary reconstructions of flat panel volume computed tomography, *HRCT* high-resolution computed tomography, *MRI* magnetic resonance imaging, *MSCT* multislice computed tomography, *N/A* not available, *TBCT* temporal bone computed tomography

The smallest and largest cochlear sizes, as measured by the A value, are shown in Table 2 as 7.4 and 10.2 mm, respectively. The shortest and longest CDL, as indicated in Table 2, are 29 and 41.4 mm, respectively. It should be noted that the measurement of cochlear size varies depending on the radiological image modality and slice thickness [41]. When measuring cochlear size, some studies have also looked at the intra- and intervariability of the software, which is roundly considered to be low [11, 38, 41, 48]. In particular, the study by Chen et al. should be highlighted, who demonstrated better internal consistency and reliability when measuring cochlear size with OTOPLAN® compared to a normal DICOM viewer [11]. Furthermore, this publication was one of the few to give a clear indication of the time to evaluate (5.9 ± 0.7 min with OTOPLAN® compared to 9.3 ± 0.7 min with another DICOM viewer).

#### Segmentation of temporal bone structures

Middle and inner ear structures including the facial nerve can be segmented and displayed in three-dimensional view (3D) in a few steps with the planning software. In this regard, four studies with OTOPLAN® are identified with our search criteria. Lu et al. reported in 2015 on 3D segmentation of the facial nerve using OTOPLAN® [47]. Compared to manual segmentation of structures, OTOPLAN® is reported to show volume differences. Andersen et al. reported segmentation of the middle ear ossicles using OTOPLAN® and compared the results with manual segmentation and automated atlas-based segmentation methods [4]. Topsakal et al. [59] and Hajr et al. [24] used OTOPLAN® to create a 3D model of the middle and inner ear structures including the facial nerve and chorda tympani. These are the reports on the application of OTOPLAN® versions 1–3. At the time of writing, version 4.0 was available, but no report on the application of version 4.0 and its accuracy in 3D segmentation of anatomical structures has been published so far. An example 3D representation of the middle ear structures and facial nerve is shown in Fig. 2d.

#### Electrode insertion trajectory and robotic drilling through the facial recess

Lovato et al. used OTOPLAN® in an ossified cochlea to visualize in the cochlear view whether the entrance of the RW is ossified or not [34]. By moving the slices up and down from the cochlear view and simultaneously checking the axial view, the presence of ossification in different planes of the cochlea can be determined. Using the segmented 3D models of the anatomical structures, the ideal electrode insertion trajectory can be planned, which passes through the facial recess, while maintaining a safe distance to the facial nerve. Topsakal et al. [58] from Belgium, Jablonski et al. [27] from Norway, and Auinger et al. [5] from Austria reported on the use of OTOPLAN® to plan safe direct cochlear access (DCA). Robotic drilling of the DCA is feasible, when following the path planned by OTOPLAN®. A slice thickness of < 0.3 mm is required for safe trajectory planning. Figure 2e shows an exemplary representation of a DCA pathway between the facial nerve and the chorda tympani with an electrode inside (From the open access publication of Jablonski et al. 2021 [27] © Jablonski et al.; CCBY4.0; https://creativecommons.org/licenses/by/4.0/).

#### Electrode position

**Fig. 3:**
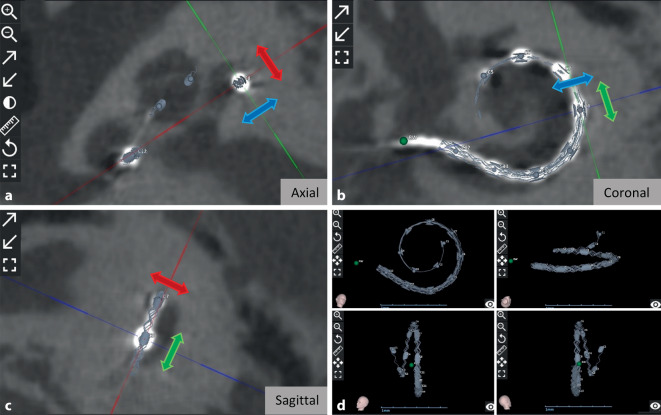
Visualization of a postoperative position control and determination of the individual electrode contacts. The evaluation in the three body planes is shown—axial (**a**), coronal (**b**), sagittal (**c**)—and the 3D representation of the implant (**d**). By moving the lines in the three body planes, each electrode can be individually controlled in the center position. *blue arrow *diameter line, *green arrow *width line, *red arrow *height line

The software can be used not only for preoperative planning of cochlear implantation, but also for postoperative localization control. In this context, five studies reported on postoperative electrode location; of these five, two were from our center [41, 42]. Dutrieux et al. [18] from France reported an electrode insertion depth (AID) of 545° with a FLEX28 electrode (MED-EL). In a small cochlea, the same electrode achieved an AID of 565°, which is only 518° in a large cochlea. Bhavana et al. [6] from India reported an average AID of 667° (range: 580–773°) with a STANDARD electrode (MED-EL). Thimsen et al. [57] from Germany reported an average AID of 663° (range: 381–798°) with a STANDARD electrode and 581° (range: 430–784°) with a FLEX28 electrode (MED-EL). Müller-Graff et al. from Germany found that the AID difference between a preoperative electrode prediction and the actual postoperative position decreases when higher-resolution imaging is used in OTOPLAN®, such as secondary reconstructions of flat panel volume CT (fpVCT_SECO_) with a slice thickness of 99 µm [42]. Figure 3 depicts the postoperative position control of the individual electrode contacts within the cochlea in the three different body planes (**a**–**c**) and in 3D visualization (**d**).

#### Patient-specific frequency map

**Fig. 4:**
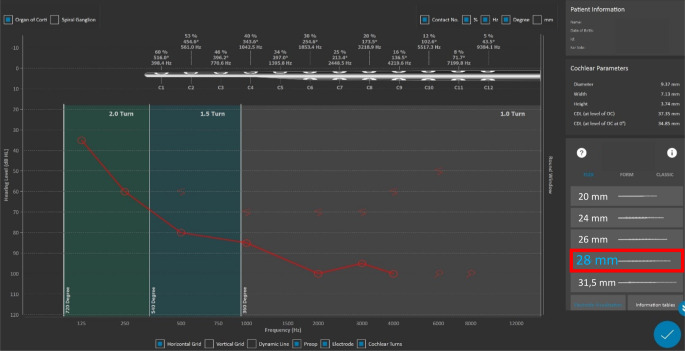
Exemplary representation of a patient-fitted electrode insertion with respect to electroacoustic stimulation

In order to further utilize the postoperative data, the software also enables the creation of patient-specific frequency maps. Di Maro et al. [17] from Italy, Mertens et al. [37] from Belgium, and Kurz et al. [29] from Germany reported the use of patient-specific (cochlear size-specific) frequency maps to minimize electrode-to-frequency mismatches. Postoperative CT scans were evaluated with OTOPLAN® to determine the array insertion depth and thus the stimulation location of each electrode in the cochlea. From the patient-specific frequency map, it appears that applying the center frequency to each stimulating electrode in combination with a longer electrode improves speech discrimination compared to the default frequency map. Figure 4 simulates a postoperative position check based on the cochlear size and shows a specific frequency assignment to each individual electrode. The frequency maps generated here can be used via additional software (MAESTRO software, MED-EL) to verify that the individual electrode contacts are within the frequency bands of the audio processor used.

#### Measurement function of temporal bone structures

**Fig. 5:**
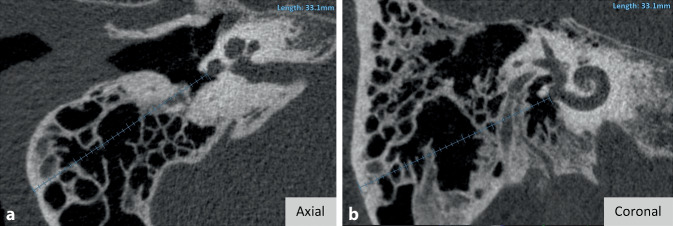
Mastoid thickness measurement on axial (**a**) and coronal (**b**) planes

The literature search resulted almost exclusively in research questions on applications of the software dealing with the cochlea. Nevertheless, OTOPLAN® also offers a measurement function for all other structures of the temporal bone. In this context, the literature search also revealed a study that applied OTOPLAN® to measure mastoid thickness and skull width in CI patients of different ages. They reported an exponential growth of both measurements until the age of puberty and almost reached a plateau thereafter [3]. The visualization of the measurement function of OTOPLAN® is exemplified in Fig. 5 using the measurement of mastoid thickness in both the axial and coronal planes.

## Discussion

OTOPLAN® is an otologic pre-planning software tool/DICOM viewer for visualizing temporal bone structures, especially the inner ear and surrounding structures. The user-friendly design simplifies the entire process of aligning images to visualize the anatomical structures of interest. A total of 32 studies, published between 2015 and 2023, were identified on the use of OTOPLAN®. Interestingly, publications with or about the software exist from many different countries and different continents, i.e., Europe, America, or Asia. The large number and worldwide distribution of papers indicates a global research interest and reflects the clinical value of this tool among clinicians in the CI field. A preponderance of studies from German-speaking countries (Germany, Austria, Switzerland: 10 of 32) is certainly due to the country of origin of the software (Austria). Nevertheless, interest in the software also seems to be increasing in locations outside Europe, such as Saudi Arabia or the United States (8 out of 32). This is presumably due to the increasing simplification of operation, the increase in useful functions of the software, and the increasing support from the manufacturing company, especially with regard to the advancement of personalized medicine.

### Assessment of the cochlear size

Of the various applications of OTOPLAN® reported in this review, 22 of 32 studies used the one assessing cochlear size. The accuracy of the oblique coronal plane in which the basal turn of the cochlea is recorded determines the accuracy of the measurement of cochlear size. The cochlear size is measured using the diameter of the basal turn from the center of the RW to the opposite lateral wall that passes through the central modiolus. This diameter of the basal turn is also referred to as the A value in CI. The CDL can then be calculated from this and sometimes other parameters (B and H values; Table 2). Since each modality uses different slice thicknesses, it is not surprising that there is some variation between the reported values. Interestingly, measurement can be made not only with CT along the bony walls of the cochlea, but also with MRI, which measures along the fluid signal of the cochlea; this seems to give comparable results [22, 60]. Thus, cochlear size can be measured not only on images from radiological devices that are radiation-based, but also from those that are not. This in turn offers enormous possibilities, especially in the implantation for children, where in the best case radiation should be completely avoided, since early exposure to radiation has been shown to lead to an increased rate of complications and long-term consequences, such as brain tumors or cataracts [44, 46]. The clinical relevance of measuring cochlear size appears to be enormous. Whereas a few years ago it was common practice to use a standardized identical electrode length for all cochleae, with the software it is now possible to select and implant electrodes adapted according to the anatomy, i.e., shorter or longer if necessary. This leads to a better mapping of the tonotopic arrangement of the sensory cells in the cochlea and, in the long run, to better hearing results [50]. Furthermore, insertion trauma, e.g., due to too-deep insertion, can be avoided by appropriate electrode selection and existing residual hearing can be preserved, e.g., with shorter electrodes. The many reliable results also indicate that the preoperative measurements of cochlear parameters with OTOPLAN® serve as a reference to answer other research questions that are not primarily concerned with the software. For example, Mlynski et al. used the preoperative OTOPLAN® data of cochlear size to show that electrically evoked compound action potentials (ECAP) are also useful for identifying postoperative electrode position [39].

### Setting the optimum measuring level

**Fig. 6:**
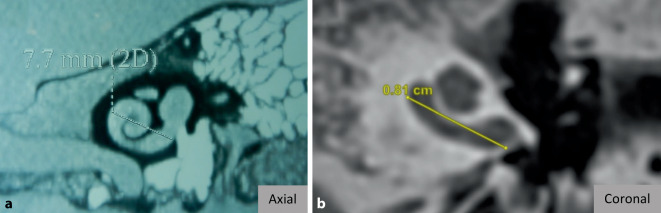
Suboptimal view of the cochlea where the size was measured in the oblique coronal plane (**a**) and in the axial plane (**b**). (**a** From Grover et al. [23], reproduced with permission © Springer Nature, all rights reserved; **b** from Mertens et al. [38], © G. Mertens et al.; CCBY4.0; https://creativecommons.org/licenses/by/4.0/)

It is evident from the literature that measuring cochlear size in a suboptimal plane, as shown in Fig. 6, only results in incorrect cochlear sizes being reported, suboptimal electrode array lengths being selected, incorrect frequency maps being created, and audio processor fitting being ineffective [23, 38]. One of the advantages of OTOPLAN® is the possibility to create the oblique coronal plane in a few steps. Here, low intra- and intervariabilities in the alignment of cochlear parameters have been shown [11, 38, 41, 48]. As an outlook, it should be noted that, in addition, the latest version 4.0 offers the possibility to automatically measure the size of the cochlea by aligning the cochlea in the aforementioned oblique coronal plane. This could ensure an even more reliable and reproducible assessment of cochlear size, although no studies with OTOPLAN version 4.0 are available yet in this regard.

### Reliability

Chen et al. reported that measuring cochlear size with OTOPLAN® had better internal consistency and reliability than using a normal DICOM viewer [11]. The time required to analyze each ear with OTOPLAN® was 5.9 ± 0.7 min compared to 9.3 ± 0.7 min with another DICOM viewer. This demonstrates the efficiency of OTOPLAN® in measuring cochlear size. In the experience of the author, who uses the software routinely, the time for the actual measurements is even shorter, in the range of 3–4 min. It is to be expected that with more frequent use, the learning curve will also increase rapidly and strongly, and thus the time required for a skilled user will decrease quickly.

### Mapping of the frequency distribution

Measurement of cochlear size enables mapping of the frequency distribution of the individual cochlea based on the Greenwood frequency function. The postoperative image provides information about the electrode insertion depth achieved during CI surgery. A combination of these two data is useful in adjusting the audio processor by assigning center frequencies to each stimulation channel based on their actual position in the cochlea. Previously, audio processors were fitted using a default frequency map [30]. Our center has investigated the hearing benefits associated with anatomy-based fitting of the audio processor based on the patient’s cochlear size. In a pilot study, this was tried in three individuals with good acceptance [29]. This indicates a great potential to perform anatomy-based fitting using the OTOPLAN software and thereby optimize hearing results. Especially for CI users with dissatisfied hearing results or other challenging cases, a new fitting, even many years after implantation, could improve CI hearing and thus further increase the acceptance of a CI.

### Planning the drilling trajectory

The entry of robotics into the CI field is to be expected in routine practice for both CI surgery and audio processor fitting. For a safe robotic drilling through the facial recess to reach the cochlea, OTOPLAN® is helpful in planning the drilling trajectory without traumatizing the facial nerve and the chorda tympani. This procedure has been successfully used by CI surgeons on more than 20 patients, with no reported case of facial nerve injury, demonstrating the effectiveness of OTOPLAN® in surveying anatomical structures [58]. Manual segmentation of anatomical structures requires patience and knowledge in order to carefully capture the structures of interest and create the 3D images. Automatic 3D segmentation of the inner ear and surrounding structures by OTOPLAN® is very convenient, especially for young and inexperienced clinicians to understand the anatomy and orientation of the structures.

### Measurement of temporal bone structures

Referring to the measurement function of temporal bone structures of the OTOPLAN software, it can be stated that this function has been used only to a manageable extent scientifically so far. One study was used to measure mastoid thickness and skull width in CI patients of different ages. Here, exponential growth of both measurements was reported until puberty [3]. Similar results are shown by Chen et al., who measured mastoid thickness without the aid of software [10]. This suggests a regular measurement function of OTOPLAN®. Overall, this function seems to have potential to support the clinician in a meaningful way, e.g., in measuring the mastoid thickness with regard to planning for the implantation of bone conduction implants.

### CI-specific DICOM viewer

CT scans of the temporal bone have been available since 1980, and there have been several research studies examining the anatomical variations of the inner ear and surrounding structures using standard DICOM viewers [53]. In the course of time, more and more approaches were developed to perform the length measurement of the cochlea on radiological images, especially mathematical methods and in the form of 3D projections [19, 26, 52]. Also, research software emerged, such as the free medical image viewers “Horos” or “3D Slicer.” These were used especially in cochlear length measurement by multiplanar reconstruction, which gave results comparable to those obtained by OTOPLAN® measurement [51]. However, there was a need for a CI-specific DICOM viewer with features that simplify the clinician’s work. OTOPLAN® is the first of its kind with CE marking to be used in clinical practice. Another recently introduced CI-specific software is the “Oticon Medical Nautilus” software (Fa. Oticon A/S, Smørum, Denmark), which also uses automated image processing [36]. However, this software is not CE certified and is currently only available as a research platform for CI-related studies. This leaves only the OTOPLAN® software as clinically applicable, which has evolved over time with good acceptance in the CI field and, according to the studies in this review, has gained worldwide recognition.

## Practical conclusion

- This comprehensive literature review included 32 studies that reported on the various applications of OTOPLAN® in the context of cochlear implantation (CI) and were published between 2015 and 2023.
- This software has been widely used for accurate assessment of cochlear size, which is known to vary in the human population. For this purpose, the highest possible image resolution, such as “secondary reconstructions of flat-panel volume CT” (fpVCT_SECO_) with 99 μm, should be aimed for clinicans, as it enables the most accurate measurements with low intra- and inter-rater variability.
- It has also been implemented in the postoperative assessment of electrode insertion depth and the application of a patient-specific frequency map in audio processor fitting. This could be of considerable relevance, particularly with regard to anatomy-based CI fitting, and could lead to an even better hearing impression in the future.
- To date, OTOPLAN® is the only CE-marked DICOM viewer for the CI field that can process pre-, intra-, and postoperative images.
- This already has and will continue to tremendously support the clinical workflow of a successful CI.

## Declarations

For this article no studies with human participants or animals were performed by any of the authors. All studies mentioned were in accordance with the ethical standards indicated in each case.

The supplement containing this article is not sponsored by industry.
